# What I Saw at Omaha1Read before the Nebraska Veterinary Medical Association, February 21, 1899.

**Published:** 1899-05

**Authors:** J. J. Drasky

**Affiliations:** Crete, Neb.


### WHAT I SAW AT OMAHA.[1]

By J. J. Drasky, V.S.,
CRETE, NEB.

I have been a member of the Nebraska Veterinary Medical Association since 1896. My reason for joining was one that possibly brought 50 per cent. of my colleagues within our circle. The only point we had in view in joining was some form of legislation benefiting and elevating the standard of the veterinarian in the State of Nebraska. With hard work and the coöperation of the entire Society we are about to take our first step by having a bill passed providing for a State veterinarian. While it may not help us all directly, we each and every one will be benefited in the end. The field is now open for operation; we are now given the opportunity wherein we can prove to the people of Nebraska that we are worthy of their confidence and protection. If the bill passes, and I believe it will, the State veterinarian and his assistants will be in a position to show to the people of Nebraska the difference between a qualified practitioner and the empiric; and, therefore, if it so happens that we hereafter may ask for some legislation along our line the task of securing it will be insignificant in comparison with our present struggles.

But I have found that there are other benefits to be derived from active membership in the Association, and of far greater importance than this. Dr. S. Stewart, in a paper read before the Missouri

[1] Read before the Nebraska Veterinary Medical Association, February 21, 1899.

Valley Veterinary Medical Association, October 5th, expresses my feelings far better than I myself am able to do. In the association we meet our colleagues, exchange with them our views on operations, if down-hearted are encouraged, and we leave for home happy, contented, refreshed, and prepared for another struggle. If we have failed in the treatment of a certain disease, we are either able to determine the cause or learn that others are not much better off than we are. We read our papers, and they are thoroughly discussed in a friendly manner, each and every one feeling perfectly at home; and I believe I am right when I say that there is not a member in our Nebraska Veterinary Association who would hesitate to speak of his non-success. Yes, I am convinced that more of us usually bring the most difficult cases, the cases which we cannot handle, before the Association, and the discussion created thereby benefits not only the individual, but the entire body. We have started in small, our membership insignificant; but while we are small in numbers we are united; each and every member is always ready to put his shoulder to the wheel and make this Association as good as the best. To illustrate the strength of union and harmony, I can only point to our meeting at Omaha. The task of entertaining the American Veterinary Association was an enormous one; still we succeeded far beyond our expectations, everything being very satisfactory, with the exception of one portion of the clinics, and the task of describing this has been assigned to me.

The clinics, as you know, are a new departure in our veterinary association, and in new features mistakes are easily made, possibly not by the entertainment committee, but, as it was with the case at Omaha, through the ignorance and previousness of someone else. I was asked by Dr. A. T. Peters to secure a ridgling horse, which I did. It was taken to Omaha by Dr. Peters with other patients, and was to have been operated on by Dr. J. W. Adams, but through some cause this gentleman was not present, and Dr. S. E. Cosford was asked to operate. There is none among us who will deny the skill of Dr. Cosford, for his long experience and success speak for themselves; but as he has been inspecting stock for the last four or five years, and, therefore, retired from active practice, it may be the cause of his partial failure, not on account of his inability, but by allowing himself to be misled by one whose conduct was anything but complimentary to the veterinary profession. I hope that none will take offense at what I may say, for it is not my desire to be sarcastic, ridicule, or speak in an unfriendly manner; but if inappropriate conduct of any of us at the Veterinary Association meetings be overlooked, the result may be disastrous to the Association. It is not from choice that I have written this paper, but it seems that in this case I am a subject of circumstances. As I have already stated, this horse was in my care. I was acquainted with the history of the case, which I gave at the clinic. As the operation caused a great deal of excitement, I was requested by a great number of my colleagues to report the result thereof. For the benefit of those who were not present, I shall attempt to describe the proceedings in detail.

The subject, a two-year-old colt, was brought out, cast and secured. Dr. Cosford, observing the necessary antiseptic precautions, washed and cleaned the scrotum with an antiseptic solution, and cleaned his hands thoroughly, cut through the common integument, exposing the external ring, then, following the inguinal canal, punctured the peritoneum about the internal ring and proceeded to secure the cord. So far the operation was performed in a very professional manner. Evidently he accomplished his desires, and in a short time succeeded in bringing to light something that to most of us appeared to be the cord; but through some, then unknown, cause the testicle failed to follow. Dr. Cosford worked very hard, and he should be complimented on his level head and steady nerve. When repeated attempts to bring the testicle forward failed, he asked Dr. Vincent, of Shenandoah, Iowa, who stood immediately behind him, if that was not the cord. Dr. Vincent in reply said : ''No ; anyone that has ever had the cord in his hand should know better,'' or something to that effect. Dr. Cosford at once released his hold upon the object in question, and proceeded once more to explore the '' lower regions ;'' but, in spite of it all, the same portion of the horse's anatomy was brought to light. After repeated failures, Dr. Cosford requested Dr. Vincent to put his hand in there and ascertain whether or not that was the cord. Dr. Vincent at once proceeded. After throwing off his coat and rolling up his sleeves, he took possession of the subject. Passing his hands into the canal, he uttered these words : '' In the first place, you are not working with the proper hand. You should go in with the right hand for the right seed and with the left hand for the left seed.'' Next he said: '' You have torn such a hole in here that when this horse gets up the guts will come down. The guts are now staring me in the face.'' Of course, such unprofessional and unscientific expressions took the breath out of the entire assembly. Some of the boys' hats blew off as the result of this unexpected gush of gas. He then continued and secured what looked to all of us like the cord, applied the écraseur, and removed the structure in question, claiming it to be the testicle. He proceeded at once to give us a free lecture on ridgling castration, telling us of the great, great many he had operated on, and the very, very insignificant losses he had met with. I shall let you imagine the effect that all of this had on the clinic class. The majority of us turned our backs upon the orator, and some of us proceeded at once to dissect the so-called testicle and found it to be nothing but a section of the spermatic cord. We, knowing that the testicle was not removed, knew that it must still be in the abdominal cavity. Many of my colleagues came to me and expressed the desire to kill the subject, hold a post-mortem examination, and prove the gentleman a prevaricator. To this I would not consent; but I gave them all my word of honor that if the horse died I would hold a post-mortem examination and publish the results.

The horse was led into his stall and taken care of by myself in person for two days that I remained in Omaha. Three or four days after the operation he was shipped with the other subjects operated upon to the experiment station at Lincoln, and in vain did Dr. Peters and his assistants strive to save the life of the patient. Six days after he arrived at the experiment station I received a message from Dr. Peters stating that my horse had succumbed, and asked me if I wished to be present at the post-mortem examination. I at once answered, requesting Dr. Peters not to do anything until I came. On arriving at the State Farm I found the carcass, and we at once proceeded with the post-mortem, which revealed inflammation of the entire abdominal cavity, enteritis, peritonitis, etc., considerable pus around the inguinal glands, a great deal of effusion and pus in the abdominal cavity, extensive adhesions, the peritoneum and intestines in all stages of inflammation, and the desired organ, the testicle, we found free in the epigastric region. The testicle being abnormal, we could readily see why the operators were unable to bring it to light by simple traction on the cord, it being considerably larger than the slit in the peritoneum. The serous covering was the only one which appeared normal. Inside of this we found a semi-osseous covering, rendering the organ unyielding and very hard. On cutting through this we found the hard shell to contain a great deal of fluid, which exuded at once. As cystic testicles are not uncommon, a trochar should be at hand at these operations, and the fluid drawn, which would cause the testicle to collapse. Still, if this had been done the consistency of the shell in this case would not have allowed the testicle to collapse. I have brought this specimen with me for your inspection, thinking that it will interest many.

I sincerely hope that this paper will be interpreted by the profession in the spirit in which I have written it. In it I reprimand Dr. Vincent, not because of any personal grudge or dislike, but for the good of the profession. I am convinced that Dr. Vincent's conduct was due to ignorance rather than to a desire to injure anyone; neither do I believe he wished to deceive us when he stated that he had removed the testicle. But in reprimanding him we warn those who may, through malice or otherwise, take an opportunity thus offered to belittle their colleagues, thus injuring the meetings, from the fact that many of those well informed lack courage and fluency of speech, and if they be handicapped by some individual possessing the gift of gab the profession is robbed in this way of valuable ideas and, possibly, of a desirable member. If he be allowed to go on in this way at our Association meetings, what will he do in active practice? How is he likely to talk to his clients, and what will his conduct be toward his competitors? I beg pardon of Dr. Vincent that it was his misfortune to be made an example of, and I assure him that I feel as friendly toward him to-day as I do to any member of the profession. I have not chosen this paper, but, as I have said before, it was assigned to me for the reason that I was responsible for the subject operated upon. I naturally became more interested than others, and our officers were justified in assigning me this paper.

